# Unraveling impaired awareness: experiences of people with dementia, Huntington’s disease and Korsakoff’s syndrome, and their informal caregivers

**DOI:** 10.1186/s12877-025-06680-4

**Published:** 2025-12-22

**Authors:** Hester Fidder, Esther de Groot, Marike E. de Boer, Marina R. Ekkel, Ruth B. Veenhuizen, Martin Smalbrugge, Anouk M. van Loon

**Affiliations:** 1 Department of Medicine for Older People, Amsterdam UMC, Postbus 7057, Location Vrije Universiteit Amsterdam, De Boelelaan 1117, Amsterdam, 1007 MB The Netherlands; 2 Amsterdam Public Health, Aging & Later Life, Amsterdam, the Netherlands; 3 Zorggroep Atlant, Apeldoorn, The Netherlands

**Keywords:** Awareness, Anosognosia, Insight, Dementia, Huntington’s disease, Korsakoff’s syndrome, Alcohol-Related cognitive disorders, Qualitative research, Informal caregivers

## Abstract

**Background:**

Impaired awareness occurs in people with dementia (DEM; Alzheimer’s disease (AD), including mixed AD/vascular), Huntington’s disease (HD) and Korsakoff’s syndrome (KS). We explored the experiences of these people and their informal caregivers, to gain a better understanding of this phenomenon.

**Methods:**

We conducted 28 in-depth, semi-structured interviews with 14 dyads, consisting of people with DEM, HD, or KS who had impaired awareness, and their informal caregivers. Topics discussed included impaired awareness of diagnosis and functioning, as well as the consequences of impaired awareness. We applied inductive thematic content analysis.

**Results:**

We identified four interconnected themes: (1) how impaired awareness is expressed by people with DEM, HD, or KS; (2) how caregivers perceive impaired awareness; (3) how caregivers respond to it; and (4) its consequences. These themes applied to four domains of awareness (diagnosis, physical and cognitive functioning, social and behavioral functioning, and risks in daily activities). Generally, people with DEM, HD or KS experienced no problems arising from impaired awareness, whereas caregivers described substantial challenges. Despite heterogeneity within individuals and domains of functioning, we observed no experiences specific to any disease group (DEM, HD or KS).

**Conclusions:**

Our findings offer insight into the diverging experiences of people with impaired awareness and their caregivers. Education about impaired awareness and managing its consequences may benefit caregivers and improve care.

**Supplementary Information:**

The online version contains supplementary material available at 10.1186/s12877-025-06680-4.

## Background

People with neurological disorders, such as dementia, may have a wide range of impairments that can impact their physical, cognitive, behavioral and social functioning. Moreover, as a result of their disease, they can have impaired awareness of their own illness, or of its implications for their functioning. This may lead to diverging experiences between these people and those around them. A range of terms, including ‘unawareness’, ‘impaired insight’ and ‘anosognosia’, are used more or less interchangeably to describe impaired awareness. Clare and colleagues introduced the term ‘awareness’, defining it as a person’s ‘ability to hold a reasonable or realistic perception or appraisal of, and/or respond accordingly to, a given aspect of one’s environment, situation, functioning or performance’ [1]. We will use this definition throughout this paper. 

Impaired awareness can have significant consequences. It may lead to an overestimation of capabilities, which can give rise to a refusal of care and engagement in risky activities [2, 3]. Additionally, impaired awareness can increase caregiver burden and stress [4–7], as well as the costs of informal care [8]. Most studies about impaired awareness have focused on Alzheimer’s disease (AD) [9–12], yet impaired awareness has also been described in other forms of dementia, including vascular dementia (VD) and frontotemporal dementia (FTD) [13, 14]. In this study, we examine three diagnostic groups: (1) Alzheimer’s disease - including mixed AD/vascular dementia - hereafter ‘DEM’; (2) Huntington’s disease (HD), a progressive, autosomal dominant neurodegenerative disorder; and (3) Korsakoff’s syndrome (KS), a neuropsychiatric disorder caused by thiamine deficiency [15–17]. Although these disorders differ in terms of their underlying pathology (neurodegenerative, genetic, and thiamine-deficiency-related) and clinical course, they all share impaired awareness, cognitive impairments, neuropsychiatric symptoms (NPS) and ultimately a need for specialized long-term care. In all three diseases, impaired awareness may pose challenges for good care provision. Examining DEM, HD, and KS together extends the study of impaired awareness beyond dementia types and allows exploration of both shared and unique experiences and care challenges across distinct disease mechanisms.

Qualitative research offers insight into people’s experiences, perceptions, and behavior [18]. However, qualitative studies on impaired awareness remain limited and have focused mainly on AD [19–23]. People with mild-to-moderate AD were shown to acknowledge “having a disease”, but had only partial awareness of specific functional losses, with weak recognition of the need for help in everyday tasks, and patient-caregiver accounts frequently diverged [20, 21]. Trindade (2023) found that awareness of relationship changes was often denied, yet more often acknowledged with close others; cognitive deficits were partly acknowledged but often normalized or minimized [22]. In young-onset-dementia (YOD), Thorsen described coping strategies such as “ignoring the dementia”, suggesting that limited or selective awareness may help preserve quality of life [23]. In moderate-to-severe dementia, Clare (2008) found that awareness was also expressed implicitly in interaction, underscoring the value of observational and conversational ways to assess awareness [24].

Notably, no qualitative studies have specifically explored impaired awareness in HD or KS, nor have studies focused solely on caregivers’ experiences. However, it is especially important to take the caregiver’s perspective into account when studying impaired awareness, since patient and caregiver often have different perceptions of the same shared reality. Therefore, this study qualitatively explores the experiences of people with DEM, HD or KS and their informal caregivers regarding impaired awareness across four main domains: diagnosis, physical and cognitive functioning, social and behavioral functioning, and risks associated with daily activities. To gain a richer and more comprehensive understanding of these experiences, we use a dyadic interview approach that integrates perspectives from both parties.

## Methods

### Design

We performed a qualitative interview study with dyads comprising one person with DEM, HD or KS and their informal caregivers; each member was interviewed separately, which allowed us to compare both perspectives on the same situations and encouraged participants to discuss sensitive issues more freely. To present findings, the COREQ checklist (consolidated criteria for reporting qualitative research) was followed [25] (see Supplementary Table 1).

### Participants and setting

Healthcare professionals identified potential participants with DEM, HD or KS during routine care. Because impaired awareness was our subject of interest, we specifically sought to include those individuals for whom impaired awareness was known to play a role. This was mainly deduced from discrepancies between the stories of patients and their caregivers, rather than by use of a formal test battery. Individuals were invited if they met the following criteria: impaired awareness of their diagnosis or social, physical or cognitive functioning; the ability to provide verbal and written informed consent; and the presence of an informal caregiver willing to participate. People with DEM, HD, or KS were purposively selected by age, sex and education level and invited to participate in person. Although information on exact disease stage (e.g., CDR scores) was not available, as is common in clinical practice, all participants with DEM and HD were community-dwelling and not in need of nursing home care, indicating that they were in the mild to moderate stages of disease. Individuals with KS resided in nursing homes but were in a steady-state condition after abstaining from alcohol since admission. The DEM group consisted of people with AD (*n* = 2) or mixed dementia: people with AD and a vascular component (*n* = 2). People with DEM or HD and their primary caregivers were community-dwelling individuals, recruited through a specialized Huntington’s outpatient clinic and a memory clinic. People with a primary diagnosis of KS or another alcohol-related cognitive disorder (Wernicke encephalopathy, Wernicke-Korsakoff’s syndrome, alcohol-related dementia, and alcohol-related brain damage) and their primary caregivers were recruited from a specialized Korsakoff ward of an LTCF (Long-Term-Care-Facility). However, due to overlapping diagnostic profiles and diagnostic uncertainties between KS and other alcohol-related cognitive disorders, distinguishing between these patient groups is difficult. Therefore, we will refer to these combined conditions as KS in this paper. Including community-dwelling people with KS was not feasible because of their known care avoidance. Recruitment and analysis were simultaneously performed: after each interview, two coders reviewed the interviews against the evolving codebook to determine whether it contained new experiences or insights, resulting in new subthemes or themes. We defined data saturation as the point at which successive dyads only elaborated on existing themes, at which point recruitment was stopped.

### Data collection

People with DEM, HD or KS and their informal caregivers were interviewed by the same interviewer, with no one else present. Interviews took place in participants’ homes (DEM, HD) or in LTCFs (KS). HF and EdG conducted all interviews; both are female PhD students and residents in elderly care medicine, trained in qualitative methods. First, people with DEM, HD or KS were interviewed to ensure an open-minded and unprejudiced interview setting and to avoid the caregivers’ accounts influencing patients’ interviews. The interview started with a neutral introductory question (“can you describe an ordinary day for you?”), inviting participants to tell openly about their own activities and functioning. Interviews took place either in participants' homes (DEM, HD) or LTCFs (KS). A topic list, composed in collaboration with the whole research team, was used to guide the interviews thereafter (See Supplementary Table 2). Topics were based on previously described domains of awareness (diagnosis, physical and cognitive functioning, social and behavioral functioning, and risks) and inspired by the Patient Competency Rating Scale (PCRS) questionnaire [26]. The topic list was used more in caregiver interviews, as caregivers could be asked directly about the impaired awareness of the person they care for. Conversations were audio-recorded and transcribed verbatim. Interviews were conducted in a neutral, non-corrective manner, focusing on everyday experiences to minimize distress. To support participants with memory difficulties, people with DEM, HD or KS were provided with an oral summary immediately after the interviews to check whether they had been understood correctly, after which small clarifications or additions were occasionally included. Caregivers were provided with either a written (a one-page summary of the interview) or oral member-check, depending on their preference. No important alterations were made after these member checks. No participants dropped out during the study.

### Data analysis

We conducted an inductive thematic analysis following Braun and Clarke [27]. First, interview transcripts and summaries were repeatedly read to familiarize ourselves with the data. The dyadic approach allowed us to jointly analyze the interviews with both parties, comparing and integrating their responses. Impaired awareness became apparent either by identifying discrepancies between the accounts of people with DEM, HD or KS and their caregivers, or without a given context (e.g., when someone denied their diagnosis of DEM, HD or KS). All interview transcripts were double-coded [HF and EdG] by labeling meaningful text fragments with names or simple phrases and checked afterwards by other members of the research team [ME and AvL]. Codes were discussed until consensus was reached. A codebook and code tree were created. During and after the coding process, main themes and subthemes were identified and discussed within the whole research team for further interpretation and verification of the findings. We used MAXQDA (2022) qualitative data analysis software to support the coding process.

## Results

We reached data saturation, as defined in the Methods section, after interviews with 14 dyads (*n* = 28) of people with DEM, HD or KS and their informal caregivers (group distribution: AD/mixed = 4 dyads, HD = 5 dyads, KS = 5 dyads). Demographic and clinical characteristics of participants are shown in Table 1.

**Table 1:** Characteristics of participants

	Person with DEM, HD or KS					Caregiver			
Dyad	Diagnosis	Marital status	Sex	Age	Education*	Relationship	Sex	Age	Education level*
DEM1	AD	Married	M	84	High	Partner	F	82	High
DEM2	AD	Married	F	83	Middle	Partner	M	84	High
DEM3	AD/vasc	Married	M	78	High	Partner	F	78	Middle
DEM4	AD/vasc	Married	F	81	High	Partner	M	77	High
HD1	HD	Married	M	72	High	Partner	F	70	Middle
HD2	HD	Married	M	66	High	Partner	F	66	High
HD3	HD	Married	F	74	Low	Partner	M	74	High
HD4	HD	Married	M	67	High	Partner	F	63	Middle
HD5	HD	Married	F	69	Low	Partner	M	72	Middle
KS1	KS	Widower	M	76	Middle	Sister	F	74	Middle
KS2	KS	Single	M	66	Middle	Daughter	F	18	Middle
KS3	KS	Single	M	72	High	Son	M	36	Middle
KS4	KS	Single	F	75	High	Friend	F	74	High
KS5	KS	Single	M	62	Low	Friend	F	50	Middle

*DEM *Dementia, *AD *Alzheimer’s Disease, *AD/vasc *Mixed dementia (Alzheimer's Disease with vascular component), *HD *Huntington’s Disease, *KS *Korsakoff’s Syndrome

*Education: Low primary education/lower (preparatory) vocational education, *Middle *higher preparatory vocational/secondary vocational education, *Higher *senior general/pre-university secondary education and higher vocational education

We identified four interconnected main themes from the data: expressions of people with DEM, HD or KS (1), caregivers’ perceptions (2) and (3) reactions, and consequences of impaired awareness (4), as well as eleven subthemes (Table 2).

**Table 2:** Themes and subthemes

Person with DEM, HD or KS	Caregiver
1. Expressions *Manifestations of impaired awareness* • Denial of deficits - “*I am not ill*”* • Minimization of problems - “*It’s still very mild*” • Other attributions - “*I am just getting old*” • A sense of resignation - “*I don’t know about myself*”	2. Perceptions *Caregivers’ perceptions of impaired awareness* • Symptom of the disease - “*It’s part of the disease*” • Intentional behavior - “*I think he doesn’t want to see it*” 3. Reactions *Caregiver reactions to impaired awareness* • Avoidance - “*I don’t want to create new problems*” • Confrontation - “*I was very direct about it*”
4. Consequences *Consequences of impaired awareness* • Risks & refusal of care - “*She doesn’t accept anything*” • Frustration and conflict - “*It’s like hitting a wall*” • Withdrawal into own world - “*He’s living in his own world*”	

*Quotes provide examples of subthemes

Themes applied to all three disease groups; the process of analysis revealed no substantial or relevant differences between DEM, KS and HD. Furthermore, themes cut across the prespecified domains of awareness. Throughout the three groups, people with DEM, HD or KS sometimes expressed partial or total awareness of their deficits in certain domains, while expressing partial or complete unawareness of their deficits in other domains at the same time. Additionally, caregivers described that impaired awareness was not continuously present.

### Main theme 1. expressions of impaired awareness

People with DEM, HD or KS generally did not experience their impaired awareness and thus were unable to explicitly reflect upon their experiences. Rather than ‘experiences of impaired awareness’, we therefore identified ‘expressions of impaired awareness’ as an overarching theme in the analysis of the data of this group. Within this general theme, we identified a spectrum of four subthemes, each representing ways in which impaired awareness was expressed by people with DEM, HD or KS. These expressions ranged from (1) a denial of deficits, (2) minimizing problems and (3) other attributions to (4) a sense of resignation.

#### Subtheme 1.1 denial of deficits - “I am not ill.”

A first way in which impaired awareness was expressed was through denial of deficits. Some people with DEM, HD or KS denied having either specific symptoms or even a diagnosis of DEM, HD or KS, while their caregivers reported the opposite. Deficits were denied in various ways, for instance by emphasizing the absence of symptoms or highlighting preserved abilities. For example, a participant who had been diagnosed with DEM said:

I am not ill. I don’t feel ill. (Person with DEM (PwDEM)#1)

Additionally, a participant with HD who demonstrated choreatic movements, described his own physical functioning the following way:

I don’t have any problems with walking and things like that. (…) I could just walk for hours. Absolutely no problems with that. No. (…) Yes, I can still cycle, I have no problems with that. (Person with HD (PwHD)#4)

While his caregiver noted:

He moves a lot. He speaks unclearly. And if you walk next to him and, well, you cannot really walk next to him in a comfortable way anymore, because he keeps on colliding. His balance is shaky and, well, cycling is actually… well, I’m not sure whether that’s responsible anymore… (Caregiver HD#4)

#### Subtheme 1.2 minimizing problems - “It is still very mild.”

A second way in which impaired awareness was expressed was through minimizing either the presence or impact of deficits, by means of normalizing or generalizing them. This was illustrated by a woman with HD and obvious choreatic movements who commented:

Well, who holds their feet completely still? (…) You are moving too. (PwHD#3)

Her caregiver, however, described how she repeatedly downplayed these choreatic movements, for example by responding:

Everyone does that. (Caregiver HD#3).

Notably, none of the participants recognized themselves in the symptoms of others with the same diagnosis. Instead, participants minimized their own problems by comparing themselves to others in the more advanced stages of the disease, who were worse off:

I think all the problems are still very mild. Right? Indeed, if you look at people who are really much further along… And I mean, I shouldn’t worry so much, it’s still very mild now. (PwDEM#1)

#### Subtheme 1.3 other attributions - “I am just getting older.”

Several participants acknowledged their own symptoms, but attributed these to causes other than their condition. For example, one participant admitted to a specialized ward for KS believed the admission was due to a recent Covid-19 infection rather than the KS diagnosis. Generally, cognitive or physical complaints were often attributed to ageing:

Yes, you get older too, so you either don’t know certain things or you, yes, or you forget them or something. (PwDEM#2)

#### Subtheme 1.4 a sense of resignation – “I just don’t know about myself.”

Participants expressed a sense of resignation or indifference about their situation: *“Then I’m like*,* whatever” (PwHD#4).* Additionally, people reported that judging their own situation or functioning was hard or impossible: *“Well*,* of course I myself can’t judge what’s been ruined in me” (PwKS#2).*

As a result, they relied on others, often their caregivers, for an evaluation of their functioning. This shaped how they spoke about their daily functioning, like a participant who remarked:

You’ll have to ask her later, I can’t really, er, judge that myself. (PwDEM#3)

### Main theme 2. caregivers’ perceptions of impaired awareness

Caregivers perceived impaired awareness on a spectrum: from a part of the disease (subtheme 1) to intentional behavior (subtheme 2).

#### Subtheme 2.1 impaired awareness is a symptom of the disease - “It’s part of the disease”

When people with DEM, HD or KS saw things differently from their caregivers, certain caregivers attributed this to the disease and responded without blame. They expressed their understanding, for example:

I think I’ve always understood it, because, well… (…) some things are just incomprehensible to him, he just can’t grasp them. That’s okay. That’s just a part of his illness, so to speak. (Caregiver KS#2)

#### Subtheme 2.2 symptoms of impaired awareness as intentional - “I think he doesn’t want to see it.”

Other caregivers however, considered this behavior deliberate (*“I think he just doesn’t want to see it.*” Caregiver HD#4). They described denial and reversal of facts:

Denying things, that it’s not true or something… Whatever I say, she will always turn things around. When I say it’s green, she’ll say it’s yellow. (Caregiver HD#3)

Some called the person with impaired awareness ‘headstrong’: *“Yes*,* she might be able to [empathize]*,* but she doesn’t want to. She’s way too stubborn for that.”* (Caregiver HD#5). Moreover, certain caregivers felt the diagnosis was used as an excuse: *“(…) She was like: “I can’t do anything anymore*,* I’m ill.” Yes*,* you are ill*,* but you can still do a lot.”* (Caregiver HD#5). Other caregivers spoke of passivity or even *‘laziness*’ (Caregiver HD#2).

### Main theme 3. caregivers’ reactions to impaired awareness

We identified ‘Caregivers’ reactions to impaired awareness’ as a third overarching theme in the analysis. Caregivers described responses to impaired awareness along a spectrum from avoidance (subtheme 1) to confrontation (subtheme 2). Their reactions were shaped by factors such as their own mood (*“When I feel a bit strained myself*,* I sometimes react in a wrong way.”* Caregiver HD#1), experience and timing. For example, some noted that certain moments were more suitable than others:

You really can’t see things as they are anymore…. But it happens at specific moments, doesn’t it? If you were to draw it on a graph, you would always have these peaks. That I think “now he’s completely off track, never mind”. And the next time you can talk to him really well. (Caregiver HD#2)

Several caregivers felt their reactions improved with progressing time and experience: “*Two years ago*,* I couldn’t talk about it yet*,* but now I can. I do notice that I am handling it better. You just have to accept it*,* there is no other way*.” (Caregiver HD#5). HD caregivers who visited a peer support group told the interviewers that talking about how to deal with impaired awareness had helped them:

Yes, it is always a search for everyone, and sometimes, yes, someone says something, and you think, oh yes, well, I will apply that, or I can do something with that, but it is a real, real search for everyone. How far can I go, for the truth… and it is no use, and that is very frustrating sometimes. I sometimes compare it with when the children were small, you know, then you could try to correct their behavior, but this can no longer be corrected. (Caregiver HD#4)

#### Subtheme 3.1 avoidance - “I don’t want to create new problems.”

Caregivers described how confronting people with DEM, HD or KS about impaired awareness often led to conflicts; as one caregiver said: “*He is in extreme denial. I am never allowed to say anything about Huntington*,* or anything related to it*,* then*,* then he can become very angry*.” (Caregiver HD#4).

As a result, caregivers tended to avoid such conversations. One caregiver questioned whether he should “*point out that there is a problem that she is not experiencing? Then you have a new problem and I don’t want that either.”* (Caregiver DEM#4) Confrontations were experienced as unproductive and escalatory, and not in line with caregivers’ desire to protect the other person’s emotional well-being. Furthermore, caregivers found it difficult to balance between being honest while protecting the other person’s feelings. Many chose not to point out the decline of the person with impaired awareness, with a caregiver noting: “*I don’t want to bring him down either*.” (Caregiver HD#1). Instead, caregivers described how they adopted discreet, pragmatic adjustments (such as changing meals to avoid choking hazards) without confronting the other directly, “*in a bit of a roundabout way*.” (Caregiver HD#4).

How much freedom do you actually have, without hurting her or worrying her, or making her panic, to just bring up things like that? That, that’s the most difficult thing, I think, that you, I’ve said before, you don’t always have to tell the truth in order not to lie. But sometimes it seems like you have to keep things quiet or something, and you don’t do that deliberately, but because you actually wonder, is there any point in bringing it up? (Caregiver DEM#2)

#### Subtheme 3.2 confrontations – “I was very direct about it.”

In contrast to avoidance, certain caregivers chose to address impaired awareness directly. They prioritized honesty and clarity, like this caregiver who confronted her father with his unrealistic plans:

But I was actually very direct about it…like “Well dad, that’s just not going to work out anymore.” (Caregiver KS#2)

A caregiver explained how she still tried to make her friend with KS understand that he had an illness, by repeatedly sharing this information with him:

[Investigator]: How is confronting him for you?

[Participant]: It’s fine… It’s not nice to do, but I still try to get it into his head correctly. (Caregiver KS#5)

### Main theme 4. consequences of impaired awareness

Caregivers described three main consequences of impaired awareness: (1) refusal of care and risks, (2) frustration and conflict, and (3) withdrawal into one’s own world. Although consequences also applied to people with DEM, HD and KS, caregivers primarily reported on this subject. Consequences of the different perceptions of reality were mainly deduced from the caregiver’s perspective, because people with DEM, HD or KS did not seem to experience problems arising from their impaired awareness.

#### Subtheme 4.1 refusal of care and risks - You don’t accept anything

Caregivers reported that people with impaired awareness often judged their need for help differently and tended to overestimate their own functioning. This led to a refusal of care and, in some cases, to dangerous behavior or situations (such as unsafe walking or cycling, or leaving the gas on when cooking). The most common point of disagreement within dyads was driving. While the person with impaired awareness often felt confident to continue (“*I would just get in and drive away*.” PwDEM#1), their caregivers doubted whether it was safe:

Yes, I don’t feel safe. Because he pushes the buttons and he looks everywhere… and I’ve also noticed in his coordination that it has become like that. (Caregiver DEM#1)

#### Subtheme 4.2 frustration and conflict - “ it’s like hitting a wall”

Caregivers found it difficult that the person with impaired awareness saw things differently from them. They described offering help that was subsequently refused: “*But well*,* you don’t want anything. Suit yourself.*” (Caregiver HD#5). This was experienced as frustrating and could lead to arguments, when neither side gave in. A caregiver described this sensation *“like hitting a wall.”* (Caregiver HD#3). Caregivers tried to accept the situation:

Sometimes it makes me angry, but yes, well, that takes a lot of energy and it doesn’t change things anyway. So I try to accept it, but it’s not always easy. (Caregiver HD#4)

#### Subtheme 4.3. withdrawal into own world - “He is living in his own world.”

Caregivers described how people with impaired awareness no longer perceived the world around them as others did (“*You really don’t see things as they are anymore.*” Caregiver HD#1). Consequently, caregivers became less able to discuss problems they experienced. They felt a distance arising as the person with impaired awareness started to withdraw into his own world: “*He really lives his own life there and*,* well*,* he doesn’t really care about the rest*.” (Caregiver KS#1). This retreating was sometimes viewed by caregivers as helpful, bringing calm: “*In some way*,* I’m happy that he is wrapped in his own cocoon*,* so to say*,* and well…that is reassuring for us too*,* I have to say*.” (Caregiver KS#1). However, at the same time caregivers also experienced a consequent loss of interest or empathy from people with impaired awareness towards them. This was often described as sad, and a loss.

I think he is so concerned with himself… So he doesn’t have that empathy anymore. He wouldn’t really ask how we are doing. (Caregiver KS#5)

## Discussion

In this study, we explored the experiences of dyads of people with impaired awareness due to dementia (AD/mixed), HD or KS, and their informal caregivers. We identified four interrelated main themes: expressions of impaired awareness by participants (Theme 1), caregivers’ perceptions (Theme 2); caregivers’ reactions (Theme 3); and consequences (Theme 4). Subthemes within each theme reflect individual variation along a spectrum; not all subthemes applied to the same person, and not every individual showed impaired awareness in all domains at once. All four themes were evident across the four functional domains (diagnosis; physical and cognitive functioning; social and behavioral functioning; risks in daily activities). Patterns did not differ by diagnosis, suggesting that impaired awareness could be seen as an overarching phenomenon with opportunities for exchanging knowledge across these different brain disorders.

Other qualitative work described varied expressions of awareness in (Alzheimer’s) dementia (Theme 1). Consistent with our findings, these studies describe how people with dementia normalize, minimize, or ignore their deficits or disease, for instance by attributing difficulties to a normal ageing process [21, 22]. Our findings indicate that this pattern occurs not only in dementia, but also in HD and KS.

From a psychosocial perspective, disease-related deficits can be understood as a threat to the self and can elicit coping mechanisms such as avoidance, denial, or repression; strategies that mirror the expressions of impaired awareness we observed. These may be employed to reduce distress and may be applied intentionally or unintentionally [28, 29]. This interpretation aligns with Clare’s biopsychosocial model, which states that the expression of awareness is not only influenced by biological or cognitive factors, but also by social and environmental ones [9]. Similarly, Sabat (2002) views impaired awareness as the product of psychosocial processes interacting with cognitive impairments [30].

Caregivers did not necessarily perceive the expressions of impaired awareness (Theme 2) as coping. Although some viewed them as part of the disease, others believed that people with impaired awareness intentionally denied the situation, its implications, or their own deficits, sometimes labeling this as ‘stubborn’ or ‘lazy’ behavior. Polenick and colleagues similarly observed that some caregivers viewed the symptoms of dementia as outside the patients’ control, whereas others assumed the patients’ “deliberate” or “malicious” intent [31]. In line with this, in a study by Davis et al., caregivers described people with dementia as ‘manipulative’ or ‘deliberate’ in their behavior [32].

Additionally, we observed that caregivers responded to impaired awareness (Theme 3) in various ways, ranging from avoidance to confrontation. Previous work shows that caregivers of people with dementia may adopt multiple, parallel coping strategies in dealing with the disease [33]. Similarly, a qualitative study involving spousal carers identified ‘avoidance’ as a way of (non-)coping with HD [34]. We observed similar patterns in all three disease groups. Overall, caregivers struggled to balance honesty with avoiding confrontation when supporting individuals with impaired awareness, regardless of diagnosis.

Finally, we observed different consequences of impaired awareness (Theme 4), including patients’ refusal of help in activities of daily living, engagement in risky behavior, and conflicts and frustrations within dyads. These observations were mainly derived from caregivers’ reports, as patients generally experienced no problems arising from their impaired awareness. Using a similar approach to ours, Mårdh and colleagues investigated impaired awareness by interviewing dyads of people with AD and their spouses separately [20]. In line with our results, the authors found that the dyads differed in opinion regarding certain issues and that patients were unable to reflect and act upon the cognitive and practical consequences of their impairment. In another study, anosognosia was associated with a threefold increase in dangerous behavior in dementia [35]. Sitek and colleagues reported that impaired awareness leads to delayed diagnosis and refusal of care in HD [16]. More generally, anosognosia has been associated with increased caregiver burden in AD and HD [4, 5]. Although caregiver burden was not explored as a primary theme in this study, it was indirectly assessed through caregivers’ accounts of differences in opinion, their reactions, and the consequences of impaired awareness, which together reflect important aspects of caregiver strain.

Furthermore, caregivers described patients as living in their own world, apparently unaffected by concerns about their condition, while caregivers faced practical difficulties and reported sadness about patients’ lack of empathy and awareness. They mourned the person they once knew and felt unable to share their struggles because of impaired awareness. This pattern reflects the “loss of a shared world” in dementia, where altered perception and memory impairments disrupt connections with others [36].

### Strengths and limitations

We consider our dyadic method a strength of this study, because this approach has enabled us to identify the different experiences that people with impaired awareness and their caregivers have of their situation. Nevertheless, a possible limitation of this method is that, in using the informal caregiver as the ‘informant’ with the more objective viewpoint, we must acknowledge the likelihood of subjective biases influencing their reporting as well.

Furthermore, this study is important because we included people with three different brain disorders, which has enabled us to compare experiences of individuals with impaired awareness across diseases.

Participant heterogeneity represents a challenge of this study. First, in the KS dyads, the informal caregivers were often not partners, but more distant relations, such as relatives or friends. This may have influenced the reported experiences, as partners typically have more frequent and intimate contact in long-term care than relatives or friends of residents. However, the patterns we observed were broadly similar to those in the dementia and HD dyads. Second, standardized measures of disease stage and disease duration were unavailable. Third, the dementia group included individuals with mixed AD/vascular dementia and with AD. Taken together, these factors may reduce comparability across participants.

In addition, different member-check formats were used (oral for patients, written for caregivers). This approach was deliberately chosen to fit participants’ needs, but may have introduced minor differences in how participants reacted to the summaries. Importantly, no substantive changes were made to the data following these member checks. A final strength of our study is that it focused on different domains of impaired awareness and therefore offers a comprehensive view of this phenomenon, while other qualitative studies so far have only focused on a specific domain of awareness. For example, Trindade and colleagues explored awareness of changes in relationships [22] and of functional status [21] in dementia separately.

### Implications for practice

Recognizing and understanding impaired awareness is important because it may lead to better care provision for both patients and caregivers and provide a starting point for the enhancement of person-centered care. We recommend offering education at an early stage, which might help caregivers view impaired awareness not as deliberate behavior, but as a symptom of the disease. This could help caregivers adopt more effective coping strategies, increase understanding, and prevent frustration and conflict within dyads. Peer support groups, in which caregivers can share experiences, receive support and learn how to deal with impaired awareness, should be offered. Discussing impaired awareness with patients should be approached with caution, because it is often unclear whether patients can understand and reflect on feedback due to cognitive impairments. Furthermore, it may negatively affect their well-being, since previous studies have linked greater impaired awareness to higher quality of life in patients with AD [4], HD [37] and KS [38]. However, when specific interventions are required, for instance because of safety risks, strategies such as ‘divergence’ rather than confrontation may be used [39].

## Conclusion

In conclusion, our findings highlight the different experiences of impaired awareness and its consequences. No experiences could be specifically related to dementia, HD or KS, suggesting that impaired awareness might be seen as a disease-overarching phenomenon. Offering education about impaired awareness, guidance on managing its consequences, as well as tailored support, may help informal caregivers.

## Supplementary Information

Supplementary Material 1.

Supplementary Material 2.

## Data Availability

The data that were generated and analyzed during the study are available from the corresponding author, upon reasonable request.
