# Supplementary material for: Unraveling impaired awareness: experiences of people with dementia, Huntington’s disease and Korsakoff’s syndrome, and their informal caregivers
Source: BMC Geriatr. 2025 Dec 22;26:55. doi: 10.1186/s12877-025-06680-4 (PMC12805700; doi:10.1186/s12877-025-06680-4)
Supplement: Supplementary file 2 — Supplementary Material 2. [file 12877_2025_6680_MOESM2_ESM.docx]

**Appendix 2: Topic List.**

Table 2. Topic List.

| ***Topic list (patient and caregiver)*** |
| --- |
| ***Opening question***  *Can you tell me what an ordinary day is like for you?* |
| ***Awareness of diagnosis (****dementia, HD or KS)*   - *Recognition of symptoms* - *Comparison to others with the same diagnosis* |
| ***Awareness of physical and cognitive functioning***   - *Changes in functioning as a result of the disease* - *Influence of disease on daily life* - *Need for help with tasks of daily living* - *Acceptance of help* |
| ***Awareness of social functioning and behavior***   - *Changes in behavior as a result of the disease* - *Changes in social relations as a result of the disease* - *Empathy* |
| ***Awareness of dangers and risks***   - *Capability for risk estimation* - *Occurrence of dangerous situations* - *Autonomy* |
| ***Impaired awareness***   - *Differences in opinion about diagnosis/physical, cognitive or social functioning, behavior and risk estimation* - *If present: reactions and consequences to impaired awareness of these domains* |
